# POOR SCHOOL PERFORMANCE: IMPORTANCE OF CHILD SELF-REPORT

**DOI:** 10.1590/1984-0462/;2017;35;4;00017

**Published:** 2017

**Authors:** Mauro Muszkat

**Affiliations:** a Núcleo de Atendimento Neuropsicológico Infantil Interdisciplinar do Departamento de Psicobiologia da Universidade Federal de São Paulo - São Paulo, SP, Brasil.

We live in a time of amazing speed in the production, diffusion and interconnection of knowledge. This rapid pace has transformed the way children think, learn, and interpret information. The school, a space for formal learning, takes a strong sociocultural and affective role in this context, particularly in Brazil, a country with so many inequalities. Thus, the role of using current teaching methods -, which allow for the improvement of skills, development of individualized and motivating teaching strategies and potentialities - also compensates for differences, reducing the inequality of opportunities. In this sense, it is key to strengthen and identify weaknesses early on, and to develop the confronting potential of the most vulnerable children. Hence the need for extensive interdisciplinary approaches to health and education professionals, both for the identification and selection of effective rehabilitation strategies in face of learning difficulties.

It is estimated that around 15-20% of children at the beginning of schooling have difficulties in learning, and these estimates can reach 30 to 50% if the first six years of schooling are analyzed. Poor school performance, defined as a lower-than-expected academic achievement for a given age, cognitive abilities, and schooling, is complex and multifactorial. It includes Learning Difficulties related to multiple factors - such as low socioenvironmental opportunities and emotional conflicts - to Learning Disorders of a neurobiological nature - such as dyslexia, dyscalculia and Attention Deficit/Hyperactivity Disorder. Poor school performance, regardless of etiology, affects the various spheres of the child’s relational network, leading to emotional problems such as anxiety, low self-esteem, and demotivation.

Recognizing how the child assesses their own performance and level of satisfaction with life starts with the metacognitive premise that the developing child is able to express themselves and access their subjectivity. The study “Quality of life and self-perception of health of children with poor school performance^1^” by the group of researchers from Universidade Federal de Minas Gerais, is a good representative of this premise. The research, carried out with 99 schoolchildren with poor academic performance, using the AUQEI Scale, validated in Brazil and easy to operate, is very welcome in this sense, because it goes against the current view that it is not enough to recognize and treat disorders, but to bring the child’s own vision, their autonomy and health perception as an aspect to enhance the sense of belonging and identity, fundamental for inclusion through education and culture.
